# A survey of availability, price and affordability of essential medicines from 2011 to 2016 in Chinese secondary and tertiary hospitals

**DOI:** 10.1186/s12939-018-0870-5

**Published:** 2018-10-19

**Authors:** Xiaodong Guan, Huajie Hu, Chunxia Man, Luwen Shi

**Affiliations:** 1 Department of Pharmacy Administration and Clinical Pharmacy, School of Pharmaceutical Sciences, Peking University, No. 38, Xueyuan Road, Haidian District, Beijing, 100191 China; 2 International Research Center for Medicinal Administration, Peking University, Beijing, 100191 China

**Keywords:** Essential medicines, Access, Availability, Median price ratio, Catastrophic drug expenditure

## Abstract

**Background:**

Essential medicines are those drugs that satisfy the priority health care needs of the population and help with functioning healthcare systems. Although many countries have formulated an essential medicine list, almost half of the global population still lack regular access to essential medicines. Research about the initiation of National Essential Medicines Policy in Chinese secondary and tertiary hospitals is inadequate, and the long-term effect on access after the reform is still unknown. This study’s objective was to investigate the access to essential medicines in mainland China’s secondary and tertiary hospitals.

**Methods:**

Data on the access to 30 essential medicines from China’s National Essential Medicine List were obtained from China Medicine Economic Information database covering 396 secondary hospitals and 763 tertiary hospitals. We improved the standard methodology developed by the World Health Organization and the Health Action International to measure the availability, median price ratio (MPR) and the incidence of catastrophic drug expenditure (CDE).

**Results:**

Five essential medicines had > 50% availability and the nationwide availability kept steady; availability of drugs in eastern regions of China was significantly higher than the central and western regions. The median MPR of 30 drugs nationwide kept steady approximately 5; MPR of drugs in the eastern regions was significantly higher than the central and western regions and the ratio of MPR of innovator brands to generics increased from 3.66 to 6.32 during the study period. The incidence of CDE caused by essential medicines decreased from 2011 to 2014; brand name medicines were more likely to cause CDE than generics and rural patients have a greater tendency to fall into CDE.

**Conclusions:**

After the implementation of National Essential Medicines Policy, the MPR of essential medicines was well controlled and became more affordable in the context of steady availability. This has highlighted the problems associated with region disparity and inequity between rural and urban areas in the delivery of essential medicines and sustainable mechanisms are needed to deepen the National Essential Medicines Policy in mainland China.

**Electronic supplementary material:**

The online version of this article (10.1186/s12939-018-0870-5) contains supplementary material, which is available to authorized users.

## Background

There are millions of people worldwide who face illness, disability, and death every year because of poor access to drugs [1, 2]. According to the World Health Organization (WHO), essential medicines are those drugs that satisfy the priority health care needs of the population and help with functioning healthcare systems [3]. They are selected on the basis of their efficacy, safety, cost-effectiveness, and ought to be available in proper dosage forms at all times [4]. One survey conducted by the WHO in 2013 estimates that over 10 million deaths worldwide could be avoided every year by an effective National Essential Medicines Policy (NEMP) [5]. To ensure the supply of essential medicines, the WHO and the Health Action International (HAI) has set a benchmark of 80% for medicine availability as high [6].

The access to essential medicines has been studied widely across the world since the WHO collaborated with HAI to develop a standardized method in May 2003 [7]. Although many middle-income countries have formulated an essential medicine list, almost half of the global population still lack regular access to essential medicines [8]. A secondary analysis of 36 developing and middle-income countries showed that the average availability of generic essential medicines was very low and treatments for acute and chronic illness were largely unaffordable in many countries [9]. In addition, generic essential drugs, whose prices are lower than brand name products, were still unaffordable in many developing countries [10–14].

Although China embraced the concept of essential medicines in 1979, the government did not introduce policies to address supply, use, payment as well as monitoring of essential medicines until 2009. In its most recent health-care reform (2009–2012), the Chinese government explicitly proposed the establishment of a national essential medicines system and made it one of five top priorities in the coming years. To ensure efficacy, government agencies –– including the Ministry of Health (MOH), the National Development and Reform Commission (NDRC), and seven other agencies –– have issued new essential medicines policies pertaining to selection, production and supply, use, pricing, payment, and other activities (see policy details in Additional file 1: Table S1 and Table S2).

Studies before 2009 revealed discouraging results about the availability and affordability of essential medicines [15–19]. With the introduction of NEMP in 2009, the Chinese government aimed at improving equity in health-care access and reducing patients’ medical costs in primary hospitals. After the initiation of the NEMP in 2009, a few cross-sectional surveys have been conducted in China utilizing the WHO/HAI methodology to obtain evidence about the access to essential medicines [20–23]. These studies showed that the availability of essential medicines decreased significantly after 2009 and the median price also fell while non-essential medicines saw less of a decline. After the initial reform, the NEMP was extended to secondary and tertiary hospitals, which were expected to ensure a certain use proportion of essential medicines [10]. However, research about the initiation of NEMP in secondary and tertiary hospitals is inadequate and the long-term effect on access after the reform is still unknown.

The main aim of this study was to measure the availability, price and affordability of essential medicines in mainland China from 2011 to 2016 by conducting a tracking survey based on the WHO/HAI methodology. To our knowledge, this is the first such survey reported in China from a national perspective that observed the temporal trends and regional disparity of access to essential medicines in secondary and tertiary hospitals. This research may assist government health policy-makers, researchers and practitioners.

## Methods

### Data sources

Information pertaining to the use of essential drugs, including dosage form, strength, purchase time, specification, manufacturer, and price information, was extracted from the China Medicine Economic Information database (CMEI). The CMEI, which was constructed in 1993, is a large observational database of drug procurement records that cover sample institutes of 396 secondary hospitals (accounting for 6.5% secondary hospitals in China) and 763 tertiary hospitals (accounting for 38.7% tertiary hospitals in China). All of these hospitals are public and are located in 28 provinces across mainland China (excluding Qinghai, Tibet and Hainan). All member hospitals submitted ample drug procurement records to the CMEI monthly and then the records were aggregated and standardized for researchers and policy-makers.

Meanwhile, Management Sciences for Health (MSH) provided the reference price from 2011 to 2013 of the generic medicines for calculation of median price ratio (MPR) [11]. That is to say, a MPR of 2 would mean that the local medicine price is twice the international reference price. MSH international reference prices, which are generally offered by not-for-profit suppliers to developing countries, are recommended as the most useful standard. Generally, a MPR of one or less indicates an efficient public sector procurement system.

China’s population income distribution was obtained from the China Statistical Yearbook from 2011 to 2014 [12–15]. (Additional file 1: Table S4) The China Statistical Yearbook provided information about income of urban and rural residents per capita for calculation of affordability.

### Sampling

Thirty medicines were surveyed from January 2011 to November 2016 in the database: 13 from the WHO/HAI core global and regional lists (representing medicines for common acute and chronic disorders) and 17 locally selected supplementary medicines chosen for their local importance and disease burden in China, with input from an advisory committee of practicing pharmacists, academics and experts. Of the 30 medicines surveyed, 27 were listed in the NEML database [16] and three were on medicines procurement supplementary list of several provinces. Eighteen medicines treat acute disorders, whereas 12 treat chronic disorders. Out of 10 therapeutic classes we surveyed, hypertension, rheumatoid arthritis and diabetes have the highest prevalence [17] among Chinese patients and so we chose four medicines of treatment for these three diseases to assess affordability (Additional file 1: Table S5).

### Measures and analysis

The availability of each medicine was reported as the percent availability of an individual medicine at the surveyed hospitals. As mentioned above, we included all of the strengths of sample medicines through measuring the chemical entities by smallest unit. Median availability of selected medicines was used in statistical analysis. We compared the availability for adjacent years, different regions, and that between innovator brands and generics.

The median price ratio (MPR), which represented the ratio of one medicine’s median unit price to the international reference price (IRP), was used for price evaluation. The median MPR of selected medicines was used in statistical analysis. To facilitate comparisons of reported figures, all reference prices of MSH from 2011 to 2013 were converted into Chinese Renminbi (CNY) over the same period by purchasing power parity (PPP) conversion rates [18]. For the absence of reference prices of MSH from 2014 to 2016, we used discount factor (DF) to discount all drug price from 2014 to 2016 into price in 2013 and then compared them with reference price in 2013 to calculate MPR(see calculation details in Additional file 1: Table S3) [19]. Finally, we analyzed differences between annual and regional MPR and between MPR of innovator brands, and MPR of generics.

Due to a lack of data about the average daily wage of the lowest-paid unskilled government workers (LPGWs), we used incidence of catastrophic drug expenditure (CDE) to assess affordability instead of LPGWs. CDE is a concept borrowed from catastrophic health expenditure (CHE) [20], which is widely used to describe all types of health expenditures that threaten the financial capacity of a household to maintain its subsistence needs. Different thresholds are used to define CHE in different researches. Generally, out-of-pocket healthcare payments (OOP) that comprise ≥10% of total household expenditures and out-of-pocket healthcare payments that comprise ≥40% of a household’s non-subsistence income are widely used [21, 22]. When only examining households with catastrophic out-of-pocket drug expenditures, a drug budget share equal to or greater than 10% is universally accepted [23]. Finally, we estimated the percentage of households with catastrophic drug expenditures (defined as a drug budget share of 10% or more) and used 7.5% and 12.5% to do sensitivity analysis. In this study we used drug expenditure instead of OOP to calculate CDE on the basis that in China copayment of hypertensive and diabetic outpatients is up to 85% and that outpatients almost fully cover drug expenditure for chronic diseases [24]. Moreover, reimbursement rate is slightly different among different provinces, therefore, drug expenditure before reimbursement is a better indicator for regional comparison. Given that we only included 3 chronic diseases which required lifelong treatment with medications, we calculated the daily average cost of medicine to estimate drug expenditure.

Due to the fact that urban residents earn an income exceeding that of rural residents, we have the figures of income per capita calculated separately for both residents.

## Results

### Availability

Among the 30 essential medicines, their median availability of different region from 2011 to 2016 were shown in Table 1. The nationwide availability remained steady and exceeded 50% in 2012 and 2015. The trend of availability in each area was consistent with the national trend and the availability of drugs in the eastern region was significantly higher than the central and western regions. Fifteen medicines had > 50% availability (Additional file 1: Table S5). Azithromycin ranked the highest and glyburide had the lowest availability. Another finding captured differences among drugs under the same category. The availability of Melformin, for example, which is a common diabetes drugs, was about 80% while another diabetes drugs such as glyburide had the lowest availability of 3%. But as for brand name and generic drug (Table 2), we found no significant difference.

**Table 1:** Median availability of 30 essential medicines in China from 2011 to 2016

Year	Nationwide (%)		Eastern (%)		Middle (%)		Western (%)		*P*
	Availability	Product specific Δ availability	Availability	Product specific Δ availability	Availability	Product specific Δ availability	Availability	Product specific Δ availability	
2011	46.0		55.4		41.4		44.9		0.000
2012	53.7	3.6*	60.1	4.7*	44.5	5.1*	47.3	3.5*	0.000
2013	48.4	−3.8*	58.1	−1.4*	41.8	−4.9*	42.5	−3.2*	0.000
2014	43.3	−2.7*	49.0	−4.5*	38.4	−4.5*	38.3	−1.4*	0.000
2015	50.5	4.6*	60.1	3.2*	47.7	5.6*	49.8	7.7*	0.000
2016	43.7	−5.2*	54.0	−5.6*	40.5	−3.6*	42.2	−6.8*	0.000

^*^P < 0.05

Product specific Δ availability: first calculated the differences of availability of each product between adjacent years and then chose the median value of these differences as Product specific Δ availability

**Table 2:** Results of Wilcoxon rank-sum test: difference between brand name and generic medicines median availability

Year	Innovator brand medicines		Generic medicines		*P*
	Availability (%)	Product specific Δ availability	Availability (%)	Product specific Δ availability	
2011	31.6		42.6		0.408
2012	31.9	1.2*	47.1	3.4*	0.379
2013	30.9	−1.4*	40.2	−3.4*	0.438
2014	26.9	−2.3*	35.8	−4.1*	0.552
2015	37.5	3.5*	41.1	6.2*	0.776
2016	30.6	−2.6*	33.8	−5.0*	0.756

^*^P < 0.05

Product specific Δ availability: first calculated the differences of availability of each product between adjacent years and then chose the median value of these differences as Product specific Δ availability

### MPR

The nationwide median MPR of 30 drugs kept steady around 5 from 2011 to 2015 but increased significantly in 2016 (Table 3). Additionally, we captured a different MPR of drugs in different areas. The MPR of drugs in the eastern region was significantly higher than the central and western regions. As for specific essential medicines, beclometasone received the highest MPR of 793.79 while ranitidine had the lowest MPR of 0.87 in 2011. The MPR of innovator brand medicines were significantly higher than the MPR of generic medicines all the time (Table 4). The ratio of MPR of innovator brands to that of generics increased from 3.66 to 6.32 during the study period. The result of median MPR of specific essential medicines is shown in Additional file 1: Table S5.

**Table 3:** Median MPR for essential medicines in China from 2011 to 2016

Year	Nationwide		Eastern		Middle		Western		*P*
	MPR	Product specific ΔMPR	MPR	Product specific ΔMPR	MPR	Product specific ΔMPR	MPR	Product specific ΔMPR	
2011	4.48		6.58		4.22		3.36		0.001
2012	4.20	−0.16	10.34	−0.08	3.64	−0.10	4.73	0.04	0.001
2013	4.70	−0.10	9.90	−0.20	4.58	−0.02	4.62	0.08	0.000
2014	5.42	−1.01	9.48	−0.24*	4.46	−0.10*	4.48	−0.06*	0.001
2015	4.58	−0.22*	8.76	−0.14*	4.43	−0.09*	4.40	−0.06	0.003
2016	11.68	0.04	12.97	−0.08	5.06	−0.04	5.01	0.05	0.001

^*^P < 0.05

Product specific Δ MPR: first calculated the differences of MPR of each product between adjacent years and then chose the median value of these differences as Product specific Δ MPR

**Table 4:** Results of Wilcoxon rank-sum test: median MPR for innovator brand medicines and generic medicines

Year	Innovator brand medicines (*n* = 16)		Generic medicines (*n* = 16)		MPR_IB_	*P*
	MPR	Product specific ΔMPR	MPR	Product specific ΔMPR	MPR_G_	
2011	29.51		8.06		3.66	0.000
2012	33.07	2.11	8.28	−0.02	3.99	0.000
2013	37.20	2.15	7.18	0.12	5.18	0.000
2014	36.57	−0.80*	7.05	−0.14*	5.19	0.000
2015	28.07	−0.85*	5.77	−0.16*	4.86	0.002
2016	34.76	−0.85	5.50	−0.08	6.32	0.000

^*^P < 0.05

*MPR*_*IB*_ MPR of innovator brand medicines, *MPR*_*G*_ MPR of generic medicines

Product specific Δ MPR: first calculated the differences of MPR of each product between adjacent years and then chose the median value of these differences as Product specific Δ MPR

### Affordability

Overall, the incidence of essential medicines causing CDE have decreased over time. It is clear that brand name medicines were more likely to cause CDE than generics and rural patients have a greater tendency to fall into CDE. The sensitivity analysis indicated that the results were robust for varying percent of income (Table 5).

**Table 5:** Incidence of catastrophic drug expenditure (%) of 4 essential medicines in China from 2011 to 2014

Treatment	Type	Percent of family income	2011		2012		2013		2014	
			Urban	Rural	Urban	Rural	Urban	Rural	Urban	Rural
Hydrochlorothiazide	Gs^a^	7.5%	0.0	0.0	0.0	0.0	0.0	0.0	0.0	0.0
		10.0%	0.0	0.0	0.0	0.0	0.0	0.0	0.0	0.0
		12.5%	0.0	0.0	0.0	0.0	0.0	0.0	0.0	0.0
Nifedipine	IBs^b^	7.5%	67.1	100.0	57.6	100.0	55.0	100.0	50.0	100.0
		10%	50.5	100.0	38.4	93.7	36.4	89.8	31.9	85.3
		12.5%	37.3	92.2	28.2	90.4	28.0	83.3	24.2	77.6
	Gs	7.5%	6.3	47.9	3.6	40.6	1.9	31.9	0.3	31.0
		10.0%	4.5	31.9	0.8	26.9	0.0	24.0	0.0	22.0
		12.5%	2.6	25.9	0.0	24.8	0.0	16.7	0.0	12.2
Diclofenac	Gs	7.5%	22.6	78.3	17.7	73.1	14.8	60.5	13.9	63.6
		10.0%	15.2	66.8	11.3	60.5	8.2	45.7	7.5	41.2
		12.5%	11.2	54.7	7.5	54.7	4.2	36.2	3.7	33.8
Metformin	IBs	7.5%	36.0	88.8	28.0	83.8	28.0	77.6	22.0	76.9
		10.0%	21.7	80.9	17.1	74.4	18.0	67.7	14.5	58.4
		12.5%	2.4	25.4	0.0	22.8	0.0	0.0	0.0	0.0
	Gs	7.5%	6.0	46.9	2.6	36.9	0.7	28.0	0.0	27.4
		10.0%	4.2	31.1	0.0	24.7	0.0	22.1	0.0	20.7
		12.5%	16.1	69.0	12.1	69.0	12.0	55.6	9.3	45.6

^a^*Gs*: Generics

^b^*IBs*: Innovator brands

## Discussion

Our findings captured some changes of trend for availability, MPR and affordability in Chinese secondary and tertiary hospitals.

Contrary to the decreasing trend for availability of essential medicines in a contemporaneous study [23], we did not note substantial decreases in medicine availability during the research period. The nationwide availability was approximately 50% from 2011 to 2016 and far from the standard of 80% set by the WHO. Some possible reasons for low availability include manufacturers’ inadequate incentives for producing essential medicines whose price was set too low [25, 26]. Additionally, from the standpoint of patients and practices, the perception that low cost essential medicines are lower quality hindered the use of essential medicines [27] and finally caused essential medicines to go out of stock in public hospitals. This phenomenon was also observed in another survey of essential medicines in Shaanxi Province in western China [28].

As for MPR, after the implementation of NEMP the rising trend was curbed until there was a sudden increase in 2016. The reasons for decrease of MPR before 2016 possibly included the pharmaceutical centralized public bidding procurement strengthened by the central government in 2014, which was a more efficient procurement to negotiate with manufacturers and wholesalers. Although the rising trend of prices of essential medicines was controlled efficiently before 2016, the MPR of 25 essential medicines still exceeded the reasonable standard of 1.5, which might be associated with disordered medicine distribution system [5, 29]. As for the increase in 2016, the most possible reason may be the withdraw of price regulations in June 2015. In September 2009, the National Development and Reform Commission (NDRC) issued regulated retail prices for essential medicines, lowering the regulated prices by 45% resulting in an average drop of 12% relative to market prices. The prices of essential medicines picked up rapidly after 6-years of price controls.

The reported affordability was also well controlled and the incidence of CDE decreased. That might primarily be due to rising living standards and increasing insurance coverage. A significant number of studies on economic reforms over the past three decades in China have identified that falling unemployment and rising real wages reduced income poverty and provided a substantial boost to household incomes [30], which enable people to afford essential medicines and high medical expenditure better.

Besides findings related to changing trend, we also found some differences among different regions, between rural and urban areas as well as generics and innovator brands.

The regional disparity of availability and MPR is of great concern. That is to say, the eastern region showed a higher availability and MPR, which might be due to the gaps in economic level and transport system among the eastern, central and western regions [31]. As extant literature documented, the central and western regions have inadequate health resources and lack high-quality essential medicines compared with the developed eastern region [23], which indicates that China is faced with a daunting inequality in health resources allocation and health services utilization [32].

Due to differences in economic development and income level between rural and urban areas, essential medicines were more unaffordable for rural patients. As for the basic medical and health services in rural areas in China, there are still issues such as health financing structural imbalance, primary health personnel deficit, irrational allocation of health resources and government funding shortage [33], which possibly hindered the affordability of essential medicines in rural areas. Medical expenditure has clearly become a heavy financial burden in rural China and one of the major poverty generators as to why many patients do not complete the appropriate treatment [23].

As the results showed, the median MPR for innovator brand medicines was about 3.66 to 6.32 times that for generic drugs. Similar results were also found in Malaysia [34]. The main possible explanation for this could be that most innovator brands surveyed are patented or imported so that competition is limited while there is fierce competition for the generic medicines because of abundant domestic manufacturers [35]. However, we did not observed any significant difference between availability of generics and innovator brands. The main reason may be that hospitals in China can only purchase two strengths for one dosage form drug and that they tended to procure one generic and one innovator brand of same generic name drug. Admittedly, a stronger evidence and a deeper analysis are needed to support the observation for further research.

Based our findings, we recommend more specific guidance for the use of essential medicines to place emphasis on prescriptions of essential medicines and improve access and equity. To secure the availability of essential medicines, investigations are also needed to evaluate the transparency and efficiency of the essential medicine bidding system among different regions. Regarding the sudden increase of MPR in 2016, price regulations of essential medicines should be strengthened and a dynamic monitoring system of essential medicine price is needed to guarantee access to affordable essential medicines.

### Limitations

As mentioned above, we improved the WHO/HAI survey manual and exceeded the specific strengths list so that the nationwide availability outclassed that of other contemporaneous research in China. Yet the study has three limitations. First, we found significant differences among drugs under the same category for MPR and availability. That is to say, we may exaggerate or underestimate the severity of the condition if we only assess the access to one drug in our survey list neglecting other therapeutic alternatives. What is more the results will be worse if we take in account other expenses such as consultation fees and diagnostic tests. Therefore, further studies can focus on assessing the access to essential medicines from the perspective of the curing process of disease instead of limited sample drug and include the alternative drugs. Second, although China’s population income curve is a good proxy for estimating income, there is still a narrow gap between the income curve from the China Statistical Yearbook and the real income distribution in China. Third, we used three parameters –– availability, prices and affordability — to reflect access to essential medicines. Other barriers, not mentioned in this work, may impair or diminish the population’s access to medicines and also need concerns for further study.

## Conclusion

This article showed that the MPR of essential medicines was controlled well after the implementation of NEMP, which have become more affordable in the context of rapid economic growth in China. Typical of most emerging economies, China faces a major challenge of region disparity and differences between rural and urban areas in the delivery of essential medicines. There should be a greater concern about equity in the allocation and efficient use of healthcare resources.

## Additional file

Additional file 1: Policies on essential medicine in China and supplementary data. (DOCX 54 kb)

## Abbreviations

CDE: Catastrophic drug expenditure
CHE: Catastrophic health expenditure
CMEI: China Medicine Economic Information database
CNY: Chinese Renminbi
DF: Discount factor
HAI: Health Action International
IRP: International reference price
LPGWs: Lowest-paid unskilled government workers
MPR: Median price ratio
MSH: Management Sciences for Health
NEML: National Essential Medicine List
NEMP: National Essential Medicines Policy
OOP: Out-of-pocket healthcare payments
PPP: Purchasing power perity
WHO: World Health Organization
