# Supplementary material for: A survey of availability, price and affordability of essential medicines from 2011 to 2016 in Chinese secondary and tertiary hospitals
Source: Int J Equity Health. 2018 Oct 19;17:158. doi: 10.1186/s12939-018-0870-5 (PMC6194621; doi:10.1186/s12939-018-0870-5)
Supplement: Supplementary file 1 — Policies on essential medicine in China and supplementary data. (DOCX 54 kb) [file 12939_2018_870_MOESM1_ESM.docx]

Table S1 New policy on essential medicine in 2009

| Policies | Objectives | | |
| --- | --- | --- | --- |
|  | Access | Quality | Rational use |
| - Administration of National Essential Medicines List (Provisional) |  | 🗸 |  |
| - National Essential Medicines List (primary care section) | 🗸 | 🗸 | 🗸 |
| - Guidance on Retail Price of National Essential Medicines (primary care section) |  | 🗸 | 🗸 |
| - Opinions on the Procurement and Distribution of National Essential Medicines (provisional) |  | 🗸 | 🗸 |
| - Regulations on Strengthening Essential Medicines Quality Supervision | 🗸 |  |  |
| - Guidance on Clinical Application of National Essential Medicines (primary care section) | 🗸 | 🗸 |  |
| - National Essential Drug Formulary (primary care section) | 🗸 | 🗸 | 🗸 |

Table S2 New managements of essential medicine in 2009

| Essential medicine | Managements |
| --- | --- |
| Selection | In August 2009, the Ministry of Health (MOH) organized experts to compile a new National Essential Medicines List (primary care section). The experts selected drugs based on clinical necessity, safety, efficacy, affordability and ease of use. Western and Traditional Chinese Medicines received equal consideration. Three hundred and seven generic drugs made the list – 205 western medicines and 102 TCM. It will be revised every 3 years. |
| Price regulation | In September 2009, the National Development and Reform Commission (NDRC) issued regulated retail prices for essential medicines, covering a total of 2349 products comprising fewer than 296 generic names. Relative to market prices, 45 per cent of the regulated prices were lower, with an average drop of 12 per cent; 49 per cent remained the same; and for 6 per cent, rare medicines, the regulated prices increased. Price regulation has been in effect since October 2009. |
| Procurement and distribution | The latest health-care reform plan proposes that essential medicines used by state-owned medical institutions should be procured by open bidding. Provincial governments would each select a single distributor. |
| Safety supervision | The China Food and Drug Administration (CFDA) now requires at least two inspections per year at essential medicines manufacturers. Essential drug manufacturers must join a centralized pharmaceutical electronic supervision network, attaching a standardized barcode to the smallest drug package. |
| Clinical use | To improve clinical use of essential medicines, the MOH issued Clinical Guidance on National Essential Medicines and National Essential Drug Formulary in December 2009. The Guidance, covering 18 common diseases encountered in primary care settings, specifies how prescribing physicians should use essential medicines based on a clear diagnosis. The formulary covers 24 treatment categories. |

Table S3 calculation of median price ratio (MPR)

| medicine’s median unit price (MP) | international reference price (IRP) | median price ratio (MPR) |
| --- | --- | --- |
| MP_2011_ | RPMSH_2011_/ PPP_2011_ | MPR=MP/IRP |
| MP_2012_ | RPMSH_2012_/ PPP_2012_ |  |
| MP_2013_ | RPMSH_2013_/ PPP_2013_ |  |
| MP_2014_*DF_2014_ | RPMSH_2013_ |  |
| MP_2015_ *DF_2015_ | RPMSH_2013_ |  |
| MP_2016_ *DF_2016_ | RPMSH_2013_ |  |

RPMSH: reference prices of Management Sciences for Health,

PPP: purchasing power parity, PPP_2011_=3.51; PPP_2012_=3.53; PPP_2013_=3.55

DF: discount factor, DF_2014_= Price_2013_/ Price_2014_=0.9833;

DF_2015_= Price_2013_/ Price_2015_=0.9602;

DF_2016_= Price_2013_/ Price_2016_=0.9350

Table S4 Description of income in China

| Year | Proportion | | Daily disposable income per capita(Yuan) ^1^ | | |
| --- | --- | --- | --- | --- | --- |
|  |  | | Urban |  | Rural |
| 2011 |  | |  |  |  |
|  | | MLDS^2^ | 8.01 |  | 4.77 |
|  | 20% | | 24.08 |  | 5.48 |
|  | 40% | | 39.72 |  | 11.66 |
|  | 60% | | 53.55 |  | 17.01 |
|  | 80% | | 72.38 |  | 24.37 |
|  | 100% | | 128.82 |  | 45.98 |
| 2012 |  | |  |  |  |
|  | | MLDS | 11 |  | 5.67 |
|  | 20% | | 28.37 |  | 6.35 |
|  | 40% | | 45.92 |  | 13.17 |
|  | 60% | | 61.42 |  | 19.29 |
|  | 80% | | 81.68 |  | 27.79 |
|  | 100% | | 140.98 |  | 52.08 |
| 2013 |  | |  |  |  |
|  | | MLDS | 12.43 |  | 6.67 |
|  | 20% | | 27.11 |  | 7.08 |
|  | 40% | | 48.30 |  | 15.11 |
|  | 60% | | 66.23 |  | 21.76 |
|  | 80% | | 89.35 |  | 31.16 |
|  | 100% | | 158.25 |  | 58.28 |
| 2014 |  | |  |  |  |
|  | | MLDS | 13.7 |  | 7.61 |
|  | 20% | | 30.74 |  | 7.58 |
|  | 40% | | 53.84 |  | 18.09 |
|  | 60% | | 73.02 |  | 26.04 |
|  | 80% | | 97.62 |  | 36.85 |
|  | 100% | | 168.81 |  | 65.61 |

*1. China Statistical Yearbook 2011～2014*

*2.* *MLDS, Minimum living daily standard*

Table S5 Availability (%) and MPR of 30 essential medicines in China from 2011 to 2016

| Drug | 2011 | |  | 2012 | |  | 2013 | |  | 2014 | |  | 2015 | |  | 2016 | |
| --- | --- | --- | --- | --- | --- | --- | --- | --- | --- | --- | --- | --- | --- | --- | --- | --- | --- |
|  | Availability (%) | MPR |  | Availability (%) | MPR |  | Availability (%) | MPR |  | Availability (%) | MPR |  | Availability (%) | MPR |  | Availability (%) | MPR |
| Sulfamethoxa-  zole(oral)^Ⅰ^ | 16.5 | 2.40 |  | 17.9 | 1.28 |  | 13.6 | 1.18 |  | 11.3 | 1.34 |  | 13.4 | 1.36 |  | 10.6 | 1.40 |
| Ciprofloxacin  (oral)^Ⅰ^ | 45.0 | 2.42 |  | 43.1 | 2.32 |  | 37.6 | 1.25 |  | 31.8 | 1.26 |  | 34.8 | 1.05 |  | 29.6 | 1.08 |
| Amoxicillin  (oral)^Ⅰ^ | 52.8 | 2.73 |  | 55.6 | 2.27 |  | 49.9 | 3.56 |  | 44.1 | 3.58 |  | 52.2 | 3.58 |  | 45.4 | 1.95 |
| Acyclovir  (oral)^Ⅰ^ | 58.7 | 3.97 |  | 62.5 | 3.80 |  | 58.4 | 3.30 |  | 55.6 | 7.26 |  | 59.2 | 3.05 |  | 56.2 | 1.60 |
| Ceftriaxone Sodium  (parenteral)^Ⅰ^ | 64.3 | 5.00 |  | 66.9 | 5.06 |  | 61.7 | 5.11 |  | 54.9 | 5.02 |  | 56.1 | 4.88 |  | 51.7 | 4.51 |
| Cefuroxime Sodium  (parenteral)^Ⅰ^ | 57.5 | 6.42 |  | 65.4 | 9.75 |  | 60.1 | 11.01 |  | 57.7 | 11.02 |  | 65.2 | 11.22 |  | 51.0 | 11.73 |
| Fluconazole  (oral)^Ⅰ^ | 74.2 | 12.74 |  | 77.5 | 17.82 |  | 72.3 | 10.91 |  | 69.8 | 15.25 |  | 72.6 | 15.03 |  | 66.8 | 14.64 |
| Azithromycin  (oral)^Ⅰ^ | 86.4 | 30.59 |  | 87.3 | 34.42 |  | 85.0 | 34.39 |  | 80.3 | 36.82 |  | 83.1 | 33.01 |  | 80.9 | 138.07 |
| Metronidazole  (oral)^Ⅱ^ | 70.2 | 1.94 |  | 76.7 | 1.60 |  | 72.7 | 1.30 |  | 67.3 | 1.13 |  | 75.4 | 1.14 |  | 66.6 | 1.25 |
| Allopurinol  (oral)^Ⅲ^ | 29.9 | 0.91 |  | 36.2 | 0.75 |  | 28.0 | 0.51 |  | 21.6 | 0.73 |  | 28.8 | 1.15 |  | 28.5 | 1.59 |
| Diclofenac  (oral)^Ⅲ^ | 62.7 | 37.78 |  | 68.6 | 40.47 |  | 64.0 | 39.91 |  | 62.3 | 42.86 |  | 65.2 | 38.23 |  | 58.4 | 37.24 |
| Aspirin(oral)^Ⅲ^ | 75.0 | 102.61 |  | 83.0 | 92.96 |  | 75.4 | 108.31 |  | 76.0 | 104.85 |  | 82.2 | 101.56 |  | 73.8 | 104.75 |
| Phenytoin Sodium  (oral)^Ⅳ^ | 17.6 | 1.13 |  | 17.0 | 1.14 |  | 16.6 | 0.91 |  | 15.1 | 0.95 |  | 17.2 | 1.25 |  | 13.9 | 1.32 |
| Carbamazepine  (oral)^Ⅳ^ | 43.1 | 2.53 |  | 44.5 | 2.36 |  | 43.2 | 2.30 |  | 38.8 | 5.07 |  | 46.9 | 2.54 |  | 42.0 | 11.64 |
| Diazepam(oral)^Ⅳ^ | 45.3 | 5.88 |  | 53.9 | 4.60 |  | 50.8 | 4.29 |  | 45.8 | 4.22 |  | 57.8 | 4.27 |  | 51.3 | 4.24 |
| Amitriptyline  (oral)^Ⅴ^ | 10.1 | 6.92 |  | 12.8 | 5.39 |  | 11.6 | 5.77 |  | 11.0 | 5.77 |  | 13.5 | 5.64 |  | 8.2 | 5.49 |
| Fluoxetine(oral)^Ⅴ^ | 30.2 | 72.79 |  | 32.7 | 126.27 |  | 32.1 | 115.67 |  | 25.5 | 113.74 |  | 31.0 | 111.07 |  | 25.3 | 108.15 |
| Atenolol(oral)^Ⅵ^ | 7.5 | 3.52 |  | 9.0 | 3.28 |  | 6.4 | 2.97 |  | 7.0 | 2.41 |  | 7.4 | 2.70 |  | 5.4 | 2.85 |
| Captopril（oral）^Ⅵ^ | 37.5 | 1.42 |  | 43.8 | 1.89 |  | 40.1 | 1.73 |  | 32.2 | 1.70 |  | 39.7 | 2.02 |  | 36.2 | 18.95 |
| Digoxin (oral)^Ⅵ^ | 37.5 | 2.28 |  | 40.8 | 2.02 |  | 42.6 | 2.18 |  | 32.6 | 2.42 |  | 37.8 | 2.05 |  | 39.7 | 36.07 |
| Simvastatin(oral)^Ⅵ^ | 62.7 | 17.65 |  | 69.0 | 12.57 |  | 60.0 | 14.94 |  | 62.0 | 19.31 |  | 66.8 | 17.84 |  | 58.8 | 17.41 |
| Amlodipine(oral)^Ⅵ^ | 71.9 | 35.08 |  | 76.5 | 36.53 |  | 73.6 | 16.03 |  | 71.3 | 15.76 |  | 79.4 | 15.24 |  | 73.4 | 14.62 |
| Nifedipine(oral)^Ⅵ^ | 80.1 | 27.44 |  | 85.9 | 48.71 |  | 83.7 | 52.38 |  | 83.8 | 51.25 |  | 85.6 | 50.05 |  | 80.4 | 48.17 |
| Beclometasone  (parenteral)^Ⅶ^ | 9.1 | 2.72 |  | 10.3 | 3.44 |  | 8.1 | 587.95 |  | 5.6 | 578.12 |  | 6.0 | 564.46 |  | 6.9 | 551.58 |
| Salbutamol  (parenteral)^Ⅶ^ | 57.7 | 793.79 |  | 63.4 | 748.54 |  | 62.7 | 741.88 |  | 60.4 | 499.29 |  | 66.0 | 365.71 |  | 62.2 | 14.64 |
| Ranitidine(oral)^Ⅷ^ | 31.6 | 0.87 |  | 34.9 | 0.78 |  | 29.8 | 0.84 |  | 23.8 | 0.82 |  | 26.8 | 0.83 |  | 22.4 | 0.97 |
| Omeprazole  (oral)^Ⅷ^ | 46.8 | 34.44 |  | 53.6 | 38.90 |  | 46.8 | 39.37 |  | 42.5 | 28.42 |  | 48.7 | 26.74 |  | 42.0 | 32.55 |
| Hydrochloro-  thiazide(oral)^Ⅸ^ | 29.8 | 1.97 |  | 36.4 | 1.66 |  | 35.7 | 1.56 |  | 30.1 | 1.78 |  | 38.5 | 2.13 |  | 31.5 | 2.21 |
| Glyburide(oral)^Ⅹ^ | 5.0 | 3.12 |  | 3.7 | 2.54 |  | 3.5 | 1.08 |  | 2.8 | 1.06 |  | 3.1 | 2.60 |  | 1.6 | 2.32 |
| Melformin(oral)^Ⅹ^ | 76.3 | 15.09 |  | 84.2 | 11.38 |  | 78.7 | 13.36 |  | 80.0 | 13.25 |  | 84.4 | 11.88 |  | 78.7 | 14.45 |

Ⅰ antimicrobial agent；Ⅱ antiparasitic drugs；Ⅲ NSAID；Ⅳ drug for nervous system；Ⅴ Antipsychotic drugs；Ⅵ drugs affecting the cardiovascular system；Ⅶ Respiratory drug；Ⅷ Digestive drug；Ⅸ diuretics；Ⅹ oral hypoglycemic drugs
